# Speed of processing training and depression in assisted and independent living: A randomized controlled trial

**DOI:** 10.1371/journal.pone.0223841

**Published:** 2019-10-17

**Authors:** Marianne Smith, Michael P. Jones, Megan M. Dotson, Fredric D. Wolinsky

**Affiliations:** 1 College of Nursing, the University of Iowa, Iowa City, Iowa, United States of America; 2 Department of Biostatistics, College of Public Health, the University of Iowa, Iowa City, Iowa, United States of America; 3 Department of Health Management and Policy, College of Public Health, the University of Iowa, Iowa City, Iowa, United States of America; Chiba Daigaku, JAPAN

## Abstract

Late life depression is widely associated with lower quality of life and greater disability, making it an important target for prevention. Earlier randomized controlled trials [RCTs] demonstrated that speed of processing training [SOPT] led to reductions in depressive symptoms and clinical depression in community-dwelling adults. Our purpose was to evaluate depression outcomes related to SOPT among older adults who live in supported senior living settings. This two-arm, parallel RCT included 351 participants aged 55–102 years who resided in assisted and independent settings in 31 senior living communities. Participants were randomized within sites to computerized SOPT vs. computerized crossword puzzles with a targeted dose of 10 hours of playtime at baseline plus 4 hours of booster training at five and eleven months. Depression outcomes included the 9-item Patient Health Questionnaire [PHQ-9] scores, categorical levels, and dichotomous indicators. Random effects linear mixed effect models estimated SOPT effects in intention-to-treat complete case and multiple imputation analyses. Mean age of the sample was 81.0 years, 72.2% were women, and 41.0% resided in assisted living. At baseline 65.7% had no depression [PHQ-9 scores < 5] and 6.6% had clinically meaningful depression [PHQ-9 scores ≥ 10]. At 12 months we found significantly increased PHQ-9 scores [*p* = 0.006] and categorical levels [*p* = 0.003], and higher percentages of PHQ-2 scores > 3 [p = 0.016] and major depressive syndrome [*p* = 0.045] among the assisted living SOPT group. No significant change in depression was observed in the independent living SOPT or attention control groups. In summary, the SOPT known as *Road Tour/Double Decision* significantly increased, rather than decreased, the burden of depressive symptoms among participants residing in assisted living. Given these risks, this SOPT program should be avoided among older people in assisted living settings, and other SOPT interventions should be combined with systematic depression monitoring.

## Introduction

Depression is a leading cause of disability and disease burden in late life [1], making it an important target for prevention [2]. Late life depression is strongly associated with cognitive impairment and diverse medical conditions that are common in late life [3–8]. Both sub-threshold and clinical depression are associated with greater functional impairments, reduced quality of life, and higher health care costs [9, 10]. Risk of late life depression increases with healthcare needs, and estimates suggest that depression occurs in 23% to 32% of older adults in residential senior living settings, 37% of hospitalized older adults, and 44% to 49% of nursing home residents [11–13]. Reducing the risk of depression onset and severity is critically important to maintaining overall health and well-being and averting the downward spiral of disability that it often triggers in older people. The relationship between depression and cognitive function, which is well-established but not well understood, is an area of increasing interest in late life depression prevention and treatment.

A considerable body of research examines the diverse ways in which depression and cognitive functions may interact to influence the performance of each. Depression is associated with dementia and thus has been evaluated as a predictor of mild cognitive impairment (MCI) and dementia, and of functional disability that routinely accompanies both cognitive disorders and depression [14–16]. Cognitive deficits in late life depression are another area of growing interest, including the role of cognitive changes that may influence older people’s response to both pharmacological treatments and talk therapies. In this context, processing speed and executive functions are considered important targets for remediation through cognitive training that will advance full treatment response to traditional therapies [17–19]. This approach emerges from research related to the neurobiological basis of depression, cognitive dysfunction, and the interaction between the two [20–22] that promises to illuminate the mechanism of action that will lead to targeted interventions that increase the likelihood of shorter illness duration and full symptomatic recovery. A small body of evidence also reports on the impact of cognitive training, using both in-person and computerized formats, on depression symptoms in healthy older adults [23–25]. To date, both the methods and outcomes are mixed as some report that cognitive training reduces depression symptoms while others report no effect.

Of importance to this study, a computerized speed of processing training (SOPT) intervention used in two large randomized controlled trials (RCTs) has been associated with reducing the risk of the onset of suspected clinical depression and reducing depressive symptoms among middle-aged and young-old community-dwelling participants [26–28]. These outcomes have important implications for older people residing in assisted living and related residential housing who are generally older and more medically complex than their home-dwelling peers [29, 30]. Although cognitive stimulation, training, and games are increasingly popular in senior living communities [31], to our knowledge, no RCTs involving cognitive training have been conducted in these environments. Specifically, no RCTs have explored the potential protective effects of SOPT training on depression among senior living residents.

The purpose of the study described here was to examine the effects of a SOPT intervention known as *Road Tour/Double Decision* on depression outcomes in an RCT in senior living settings. The *Improving Mood in Assisted Living Settings* (Mood Study) RCT builds on methods used in the earlier *Advanced Cognitive Training for Independent and Vital Elderly* (ACTIVE) [26, 28] and *Iowa Healthy and Aging Minds Study* (IHAMS) [27] which reported beneficial effects of the SOPT on depression outcomes. The first aim of the Mood Study was to demonstrate that *Road Tour/Double Decision* worked equivalently on cognitive speed of processing with the generally older population that resides in senior living settings [32]. The second and primary aim of the study was to evaluate the effect of *Road Tour/Double Decision* on depression symptoms and syndromes using a clinically sensitive measure of depression, the 9-item Patient Health Questionnaire (PHQ-9) [33], as outlined here. The third aim was to address factors that are highly associated with late-life depression. Given the risks associated with late life depression, the SOPT intervention presented an easy-to-use and access, low-stigma approach to reducing depression symptoms.

## Materials and methods

### Human subjects, protocol, participant enrollment, and setting

We obtained human subject approval for the Mood Study from the University of Iowa (UI; IRB Protocol 201208786). The protocol was registered with *ClinicalTrials*.*gov* in January 2013 (NCT 01763216). Enrollment occurred between May 2013 and October 2015, and all follow-ups were completed by October 2016. Because the design has been detailed elsewhere [32, 34], only a brief overview is provided here.

The study design used a community-based participation approach in which assisted living residences were invited to engage as active partners who implemented study methods in collaboration with the university team. Study methods were tailored to fit the individualized needs of senior living partners, but all followed the same basic protocol to assure fidelity to our methods. Partner organizations agreed to have one or more staff members, called Study Liaisons, trained and certified as on-site research assistants that completed study-related activities, and to engage a minimum of 10 participants in the study. These criteria promoted inclusion of geographically distant senior living programs while conserving travel time by the university team to train Study Liaisons and assist them to be successful.

The on-site Study Liaisons advertised the study, recruited volunteer older adults to participate, completed consent documents, collaborated with the University of Iowa (UI) Study Coordinator (MMD) to randomize participants, trained participants to use assigned computerized “games” (i.e., intervention and attention control software), administered on-site computer-based assessments, and collaborated with the university team to schedule telephone interviews. Partner organizations were provided honoraria to offset their study-related costs, with follow-up honoraria given after the recruitment goal of 10 participants was met or exceeded. Because of the slower than expected take-up among assisted living residents, we extended eligibility to older adults who lived at the same senior living campus, but in independent living. The main contributing factors for this modification were the inability of assisted living programs to meet participatory design demands based on their small size (e.g., did not have 10 eligible residents or sufficient staff support) and input from key leaders that older adult characteristics in assisted vs. independent living were similar [34]. Study approaches and protocols were the same across assisted and independent living programs and participants, with fidelity checks provided by the university study team.

The study used broad inclusion criteria: having sufficient vision and dexterity to use a monitor, keyboard, and mouse; capacity to sign meaningful informed consent; and being 55 years old or older. Visual ability was self-reported and checked as needed by having participants read short printed passages. Sufficient dexterity to use the computer was based on self-report and observation during the recruitment and consent process. The lower age bound (55 years) was adopted at the request of partner organizations to reflect their admission policies and thus improve inclusiveness. Additional exclusion criteria were not used because the goal was to include a diverse cross-section of older people that was comparable to earlier community-based SOPT studies. Specifically, we did not screen participants for either cognitive or depression levels. Capacity to sign meaningful informed consent served as a proxy for cognitive impairment that would interfere with completing the SOPT intervention. Given that state level assisted living policies did not allow persons with more than mild cognitive decline (score > 3) on the Global Deterioration Rating Scale [35] to reside in this setting and that cognitive status was therefore assessed annually, the use of meaningful consent was considered sufficient by all parties involved. Similarly, the presence or absence of depression was not a criterion because the focus was on depression prevention in senior living settings, not on the treatment of existing illness. To that end, advertising materials focused on evaluating computerized cognitive training that may improve mood.

UI staff conducted baseline telephone interviews using *REDCap* software [36] that averaged 35 minutes, and follow-up telephone interviews at 5–8 weeks (post-training), and at six and twelve months that averaged 30 minutes. All outcome measures were collected at each of the four time points; demographic and background information was collected once at baseline assessment. Participants were compensated for each completed interview. The computerized Useful Field of View (UFOV) test that measures cognitive processing speed [37] was separately administered on-site by Study Liaisons at the time of each telephone interview. The UFOV includes subtests for processing speed, divided attention, and selective attention that were summed to yield total time (51–1500 *ms*).

### Randomization

Individuals who signed informed consent documents were randomized to intervention or attention control groups. Sequential randomization letters were computer-generated separately for each assisted and independent living residence within each of the 31 senior living communities by the study biostatistician (MPJ). A 1:1 allocation ratio was used along with permuted blocks of size two and four. Randomization letters were securely stored in the UI Study Coordinator’s (MMD) office in opaque envelopes, which she opened only after the matching baseline interview and UFOV were completed, ensuring complete baseline blinding. UI staff were further blinded when conducting follow-up interviews.

### Intervention

We used Posit Science’s enhanced versions of the SOPT used in ACTIVE and IHAMS (https://www.brainhq.com/) [38–40]. *Road Tour* on CD was used until Posit Science unexpectedly replaced it with *Double Decision*, a web-based version that had equivalent efficacy in achieving UFOV outcomes but was easier to access and use. The unanticipated change in SOPT format was addressed by assuring that individual participants used only one version, and by confirming the equivalence in post-hoc analyses (described elsewhere) [32]. Although visually somewhat different from one another, *Road Tour* and *Double Decision* performed the same way. In its least challenging level, SOPT participants saw an object (either a car or truck) in the center of the monitor and a target (route 66 road sign) along with seven rabbit distractor signs in a near-periphery orbit. Participants viewed the monitor image as quickly as they could (measured in *ms*) while still correctly identifying the object (car or truck) and the target (route 66 sign) location. The SOPT program assessed each participant to set the initial challenge level. Challenge levels were raised by adding more distractors, moving the route 66 sign into further peripheral orbits, morphing the car and truck to become alike, and making the background image more complex. Increased challenge levels (adaptive changes) occurred only after correct identification of the car vs. truck and route 66 sign location was successful in > 75% of the trials at the current challenge level. We asked participants to complete ten hours of training within 5–6 weeks of baseline, and to complete four additional hours each at months 5 and 11. The SOPT program electronically recorded the participant’s completed time on the task.

### Attention control

Attention control participants used Boatload Puzzles, LLC’s *Boatload of Crosswords* (https://www.boatloadpuzzles.com/) that was used in earlier trials [27, 41]. These participants saw a traditional puzzle format on the monitor. Unlike a paper and pencil puzzle, *Boatload of Crosswords* participants used the mouse and keyboard to enter their answers to the row and column clues. They could also select the size and complexity of the puzzle, and use radio buttons to fill in letters or words, show incorrect entries in a different colored font, or solve the entire puzzle. Unlike SOPT, *Boatload of Crosswords* did not present greater challenges as user’s skills improved. We used the same training schedule for attention control participants, but the software did not electronically record playing time.

All participants were asked to record their playing time on individualized “time cards” to promote completing the targeted dose at each interval. Time cards were used to help participants be self-sufficient in tracking their playing time vs. asking Study Liaisons to cue, track, or monitor their time. Study Liaisons were asked to provide encouragement to play the games and to collect completed time cards, although adherence to the latter was suboptimal.

### Depression outcomes

The depressive symptoms and depression outcomes at baseline and 12 months were based on the 9-item Patient Health Questionnaire (PHQ) [33] which is routinely used in research and clinical practice to quantify late life depression and assess changes over time [42]. The PHQ-9 items reflect all of the DSM-5 diagnostic criteria for major depression [43] and have four response options ranging from 0 = not at all, 1 = several days, 2 = more than half the days, to 3 = nearly every day, for a score range of 0–27. The PHQ-9 scoring manual [44] offers several alternatives for interpreting scores, including (a) total scores on the 27-point scale, (b) score categories from none to severe; (c) PHQ-2 hallmark symptoms that signal risk of clinical depression, (d) clinically significant levels; (e) Major Depressive Syndrome, and (f) dichotomous scoring (present/absent) reported as percentages for positive PHQ-2 scores, change in PHQ-9 levels, PHQ-9 scores ≥ 10, and Major Depressive Syndrome.

In our study total PHQ-9 total scores (0–27) were reported as means. Score categories using established cut-points for clinical depression were coded as levels, with 1 = 0–4 for minimal or no depression; 2 = 5–9 for mild depression; 3 = 10–14 for moderate depression; 4 = 15–19 for moderately severe depression; and 5 = ≥ 20 for severe depression. These levels were summarized as means and percent present by level. The first two items of PHQ-9, anhedonia and prominent dysphoria, are hallmarks of major depression that can be scored independently (called the PHQ-2); scores ≥ 3 that suggest risk of major depression [44] were summarized as the percent present. PHQ-9 scores of 10 or greater indicate clinically significant depression that should be a focus of treatment were reported as percent present [44, 45]. PHQ-9 items were also scored as Major Depressive Syndrome which aligns with diagnosis of major depression (i.e., a total of five symptoms present more than half the days that includes one or both hallmark symptoms) [44] and were reported as percent present.

### Covariates

Selected covariates were included in the analytic models to ensure proper attribution of observed SOPT effects. Age (coded in years), sex (coded 1 = male, 0 = female), and education were included to adjust for sociodemographic factors. Education was assessed by asking participants their highest level of education as an open-ended question, with answers confirmed by the interviewer, and then coded for analyses (1 = none, 2 = grades 1 to 8, 3 = grades 9 to 11, 4 = high school or general education development completion, 5 = vocational or trade school, 6 = some college, 7 = college graduate, and 8 = graduate training). Education was reported as mean of the coded levels.

Health conditions and health status were included to adjust for illness burden and function. The number of comorbid conditions was assessed by reading a list of 17 common conditions which participants were asked to rate yes/no in relationship to the question “Has a health care professional ever told you that you have (name of condition). The number of comorbid conditions was summarized as a total count (0–17) and reported as a mean. One item about general health status was self-rated as 5 = poor, 4 = fair, 3 = good, 2 = very good, and 1 = excellent, and was reported as a mean.

### Hypotheses, sample size, power, and stopping rules

We hypothesized that the SOPT group would have reductions in depressive symptoms and clinical depression compared to the attention control group. Because the Mood Study was powered based on processing speed and the PHQ-9 had not previously been used with SOPT, power calculations could not be directly calculated. Nonetheless, using one-year results from ACTIVE and IHAMS for the CESD-12 [26, 27], a projected 10% attrition rate [46], and one-tailed tests of our directional hypotheses, we estimated ≥ 80% power with 300 participants at baseline. Here, however, only two-tailed *p* values ≤ 0.05 are considered statistically significant. Stopping rules were applied once half of the participants completed the study and were set at *p* < 0.003 for efficacy and at *p* < 0.10 for futility. Neither stopping rule threshold was reached.

### Analyses

Consistent with earlier SOPT trials [26–28], we focused on changes from baseline to 12 months on the PHQ outcomes. The SOPT vs. attention control groups and the assisted vs. independent living residents were compared on demographic, socioeconomic, comorbidity, and self-rated health measures (the covariates), as well as the baseline study outcomes using chi-squared and Student’s t-tests. Markov Chain Monte Carlo (MCMC) multiple imputation for missing data on the study outcomes was conducted before performing any multivariable analyses. The multiple imputation used the covariates and the baseline PHQ-9 scores. To adjust for the clustering of participants within senior living communities, intention-to-treat (ITT) analyses used random effects in linear mixed effect models (LMEMs) to predict average treatment effects (ATEs) on changes in the PHQ-9 scores and levels from baseline to 12 months, to predict the dichotomous outcomes at 12 months, and to estimate marginal means. Both complete case analysis and the MCMC imputation approach were used. Finally, to address the potential for attrition bias affecting the complete case analyses we estimated a binary logistic regression model of the complete vs. the non-complete cases. All of the variables used in our main analyses were used as the predictors, with the only significant predictor being assisted living status (*p* ≤ 0.001). Those in assisted living were much less likely to have complete data at one-year (68.5%) compared to those in independent living (86.6%). However, there were no differences in attrition rates between participants in SOPT vs. participants in the crossword puzzles groups. Thus, those in assisted living were more likely to be lost to follow-up, but were equally likely to do so whether exposed to the intervention or not. Accordingly, it is unlikely that our results are due to differential attrition. All analyses were conducted using *IBM SPSS Software*, *v25* [47].

## Results

**Fig 1:**
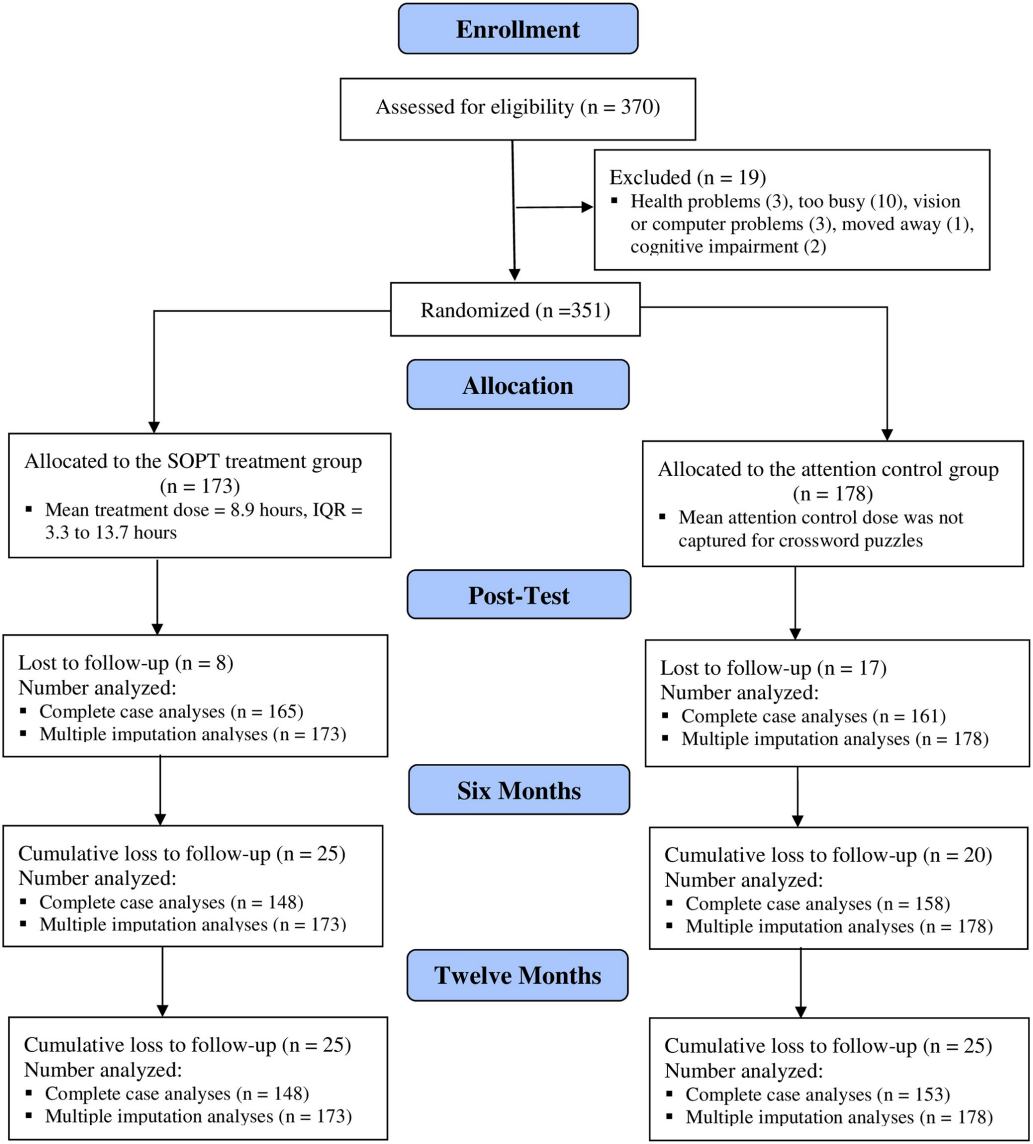
CONSORT flow chart for improving mood study.

Of the 370 consented potential participants, 19 were excluded prior to randomization [32]. We randomized 351 participants and 78.1% had complete data at 12 months (Fig 1). Among participants with complete data (n = 274), mean age was 80.6 years (*SD* = 9.1) years, 72.7% were women, 98.5% were white, and mean educational level was 5.9 (*SD* = 1.5) suggesting most had vocational/technical education or some college. The mean number of comorbid conditions identified by participants was 4.7 (*SD* = 2.4), mean self-rated health was 2.7 (*SD* = 0.9), and 41.2% resided in assisted living. By comparison, the National Center for Health Statistics (NCHS) reports that residential care communities nationwide include 52% who are aged 85 years and older, 30% who are aged 75–84 years, 10% who are aged 65–74 years, and 7% who are under age 65 (mean age is not reported). NCHS also reports that in residential care communities 70.2% are women, and 84.3% are non-Hispanic white [11]. In Iowa residential care communities 97.3% of residents aged 65 years and older are white [48].

Two group comparisons (intervention vs. attention control) revealed no significant differences between groups. By contrast, four-group comparisons (treatment group by setting type) that were undertaken to evaluate the possible impact of expanding participation from assisted to independent living identified differences between assisted and independent living participants at baseline assessment [34]. Treatment group by residence type comparisons indicated significantly higher percentage of men and lower self-rated health for those in assisted living, regardless of treatment group. Both the greater percentage of men and lower self-rated health among participants in assisted living is consistent with health-related declines that require assistance. Nationally, service needs among residential care community members include help with bathing (62%), dressing (47%), toileting (39%), transferring (30%), walking (29%) and eating (20%) [11].

The distribution of SOPT treatment dose, measured electronically, was negatively skewed resulting in a mean of 10.3 hours and *IQR* = 5.3 to 14.8 hours at study completion. Time spent using the SOPT was significantly lower for the assisted living participants (Mean = 9.8 vs. 10.7 hours, *p* = 0.417), and both were noticeably less than the targeted study dose of 18 hours. Measuring treatment dose for the attention control group using time cards proved ineffective as fidelity to collection methods was low among Study Liaisons.

Cognitive speed of processing measured by UFOV composite scores were significantly lower at baseline among participants residing in assisted vs. independent living (Mean = 609.7 *ms* vs. 425.9 *ms*; *p* ≤ 0.001). All SOPT participants demonstrated statistically significant declines in UFOV scores (e.g., faster processing speed) vs. the comparison group. Compared to earlier community-dwelling samples that used the SOPT known as *Road Tour*, however, participants in this study had lower UFOV composite scores at baseline and demonstrated lower gains over the course of training [32,34].

### Unadjusted PHQ-9 outcomes at 12 months

Table 1 contains unadjusted PHQ outcomes (PHQ-9 scores, PHQ-9 categorical levels, PHQ-2 scores ≥ 3, PHQ-9 scores ≥ 10, and PHQ-9 scores reflecting major depressive syndrome). At baseline, 65.7% of the sample had no depression (PHQ-9 scores < 5) and 27.7% had mild depression (PHQ-9 scores of 5–9) which is slightly higher than national reports that 23% of older people in residential care experience depression [11]. As with participant characteristics, four group comparisons (treatment group by setting type) were undertaken to evaluate the possible impact of expanding recruitment from assisted to independent living. This analysis indicated that participants in assisted living had higher PHQ-9 mean scores (*p* = 0.018) and PHQ-9 levels (*p* = 0.013) at baseline compared to those independent living.

**Table 1:** Unadjusted means or percentages for PHQ-9 outcomes among 274 participants with complete data at baseline and 12 months.

Measure	Speed of Processing Treatment			Crossword Puzzles Attention Control		
	Assisted Living (N = 55)	Independent Living (N = 79)	Total (N = 140)	Assisted Living (N = 58)	Independent Living (N = 82)	Total (N = 134)
**Baseline PHQ-9 Outcomes**						
PHQ-9 Score^a^	5.2	3.2	4.0	4.1	3.7	3.8
PHQ-9 Level^b^	1.7	1.3	1.4	1.5	1.4	1.4
PHQ-2 Score ≥ 3^c^	3.6%	3.8%	3.7%	6.9%	6.1%	6.4%
PHQ-9 ≥ 10^d^	12.7%	3.8%	7.5%	6.9%	4.9%	5.7%
PHQ-9 Major Depressive Syndrome^e^	3.6%	0.0%	1.5%	1.7%	3.7%	2.9%
**12 Month PHQ-9 Outcomes**						
PHQ-9 Score^a^	6.6*	3.6	4.8*	4.2	4.2	4.2
PHQ-9 Level^b^	2.0*	1.4	1.6**	1.5	1.5	1.5
PHQ-2 Score ≥ 3^c^	21.8%**	3.8%	11.2%*	12.1%	7.3%	9.3%
PHQ-9 ≥ 10^d^	21.8%	3.8%	11.2%	8.6%	9.8%	9.3%
PHQ-9 Major Depressive Syndrome^e^	14.6%*	2.5%	7.5%*	3.5%	2.4%	2.9%

*Baseline to 12 month changes are significant at *p* ≤ 0.05 as indicated by paired t-tests.

**Baseline to 12 month changes are significant at *p* ≤ 0.01 as indicated by paired t-tests.

***Baseline to 12 month changes are significant at *p* ≤ 0.001 as indicated by paired t-tests.

^a^PHQ-9 means; scores range from 0 to 27 with higher scores indicating more severe depression.

^b^PHQ-9 means; levels range from 1 (none; scores ≤ 4) through 5 (severe depression; scores ≥ 20).

^c^PHQ-2 scores ≥ 3 for hallmark symptoms of “little interest” and “feeling down” items are a positive screen; reported as percent present.

^d^PHQ-9 scores ≥ 10 indicates clinically meaningful depression; reported as percent present.

^e^Major Depressive Syndrome includes 5 PHQ-9 items that are scored “more than half the days” including one or both hallmark symptoms of “little interest” and “feeling down”; reported as percent present.

Participants in the assisted living SOPT group demonstrated the greatest change by 12 months, with PHQ-9 mean scores increasing from 5.2 to 6.6 (*p* = .006), mean PHQ-9 categorical levels increasing from 1.7 to 2.0 (*p* = .003), percent with PHQ-2 scores ≥ 3 increasing from 3.6% to 21.8% (*p* = .016), PHQ-9 scores ≥ 10 increasing from 12.7% to 21.8% (*p* = .068), and the percent with major depressive disorder syndrome increasing from 3.6% to 14.6% (*p* = .045). Participants in the independent living SOPT group demonstrated little change in depression from baseline to 12 month assessment (i.e., none were significant using paired sample t-tests). In turn, significant changes for the SOPT group as a whole (assisted and independent living groups combined) in PHQ-9 means scores (*p* = .021), categorical levels (*p* = .008), PHQ-2 scores ≥ 3 (*p* = .018) and major depressive syndrome (*p* = .011) are the result of changes among to assisted living participants. Participants in the attention control group trended toward stable depression at 12 months.

### LMEM models of PHQ-9 scores and levels

Table 2 contains the regression coefficients for the complete case analyses (comparable results were obtained from the MCMC multiple imputation approach; not shown) of the ITT random effect LMEMs of the PHQ-9 scores and categorical levels at 12 months. The predictors were SOPT vs. attention control, assisted vs. independent living, the interaction of treatment group and residence status (heterogeneity of treatment effects; HTEs), the random effects for clustering, the baseline PHQ-9 scores, and the covariates. Significant effects for SOPT, residence status, their interaction (HTE), and the PHQ-9 baseline scores were observed for both the PHQ-9 scores and categorical levels at 12 months, while the covariates did not have significant effects.

**Table 2:** Intention-to-treat results for the 274 cases with complete data using random effects linear mixed effect models to predict PHQ-9 scores and categorical levels at 12 months.

Baseline Predictors	PHQ-9 Score		PHQ-9 Categorical Levels	
	Regression Coefficient (*b*)	*P*-Value	Regression Coefficient (*b*]	*P*-Value
Intercept	-1.74	0.547	0.61	0.255
Intervention^a^	-2.10	0.001	-0.41	0.001
Residence type^b^	-1.55	0.034	-0.30	0.025
Interaction^c^	2.45	0.004	0.52	0.001
Cluster Random Effects Vector^d^	--	0.270	--	0.010
PHQ-9 Score at Baseline^e^	0.58	0.001	0.10	0.001
Age in Years	0.03	0.270	0.00	0.389
Sex^f^	-0.20	0.692	-0.10	0.282
Educational Level^g^	0.16	0.270	0.02	0.435
Comorbidity Count^h^	0.08	0.448	0.02	0.326
Self-Rated Health^i^	0.07	0.811	-0.01	0.918

^a^Intervention is coded 1 = SOPT, 0 = attention control.

^b^Residence type is coded 1 = assisted living, 0 = independent living.

^c^Interaction is coded 1 = SOPT and assisted living, 0 = all others.

^d^Vector of 30 random effect terms reflecting 31 settings/communities.

^e^PHQ-9 score is the mean score on a scale from 0–27.

^f^ Sex is coded 1 = male, 0 = female.

^g^Educational level is the mean of levels coded 1 = none, 2 = grades 1 to 8, 3 = grades 9 to 11, 4 = high school or equivalent, 5 = vocational or trade school, 6 = some college, 7 = college graduate, and 8 = graduate training.

^h^Comorbidity count is the mean of 17 health-related problems identified from the list.

^i^Self-Rated Health is the mean of one self-rated item coded 5 = poor, 4 = fair, 3 = good, 2 = very good, and 1 = excellent.

Because of the significant interaction, understanding these effects is best reflected by the estimated marginal means adjusted for the other factors in the model, as well as stratified analyses separately within assisted vs. independent living. The estimated marginal means for PHQ-9 scores at 12 months were 5.90 for those in the assisted living SOPT vs. 4.35 in the independent living SOPT groups, and 3.80 vs. 4.70 in the comparable attention control groups. Consistent with the unadjusted scores, these marginal means increased among those in assisted living exposed to SOPT, while PHQ-9 scores decreased somewhat for SOPT exposure among those in independent living. Similar results were found for PHQ-9 categorical levels, with estimated marginal means of 1.93 for the assisted living SOPT group vs. 1.53 for those in the independent living SOPT group, and 1.43 vs. 1.65 for those in the comparable attention control groups.

### LMEMs for PHQ dichotomous outcomes

Table 3 contains the estimated marginal means obtained from the complete case (again, comparable results were obtained from the multiple imputation approach; not shown) ITT random effects LMEMs of the four dichotomous PHQ outcomes at 12 months. As with Table 2, the predictors were SOPT vs. attention control, assisted vs. independent living, the interaction of treatment group and residence status (HTEs), the random effects for clustering, the baseline PHQ-9 scores, and the covariates. The interaction of treatment group with residence status (HTE) was significant for two of the dichotomous outcomes: increased percent with PHQ-9 categorical levels ≥ 1 level higher, and, in increased percent with PHQ-9 scores ≥ 10. Significantly higher percentages with worse outcomes occurred among those in the assisted living SOPT group, while SOPT exposure among those in independent living did not produce significant change.

**Table 3:** Estimated marginal means (percentages) from the intention-to-treat results from 274 complete cases using random effects linear mixed effect models^a^ to predict PHQ-9 Dichotomous Outcomes at 12 Months.

One-Year PHQ-9 Dichotomous Outcomes^b^	SOPT		Crossword Puzzles		*P*-Value^c^
	AL	IL	AL	IL	
PHQ-2 Score ≥ 3^d^	17.5%	5.1%	10.3%	9.0%	0.120
≥ 1 PHQ-9 Levels Worse^e^	36.8%	20.8%	15.7%	27.4%	0.010
PHQ-9 Score ≥ 10^f^	16.7%	10.0%	5.4%	15.0%	0.021
Major Depressive Syndrome^g^	2.3%	2.2%	3.0%	5.3%	0.366

AL is Assisted Living; IL is Independent Living; SOPT is speed of processing training, the intervention; Crossword Puzzles is the attention control.

^a^These models included the same baseline predictors as shown in Table 2.

^b^PHQ-9 outcomes were dichotomized as present or absent and are reported as percent present at 12 months.

^c^This is the probability value for the interaction of speed of processing treatment with residential status (assisted living vs. independent living) and is the test for the heterogeneity of the treatment effect (HTE) by residential status.

^d^PHQ-2 score is for “little interest” and “feeling down”; score ≥ 3 reflects a positive screen.

^e^PHQ-9 levels range from 1 (none; scores ≤ 4) through 5 (severe depression; scores ≥ 20); one level worse is change from baseline by one or more level (e.g. none = 0–4 to mild = 5–9) at 12 months.

^f^PHQ-9 scores ≥ 10 indicates clinically meaningful depression.

^g^Major Depressive Syndrome includes 5 PHQ-9 items that are scored “more than half the days” including one or both hallmark symptoms of “little interest” and “feeling down.”

## Discussion

This study was designed to examine whether the SOPT interventions known as *Road Tour/Double Decision* would have an equivalent positive effect on depression among the generally older group of individuals who have selected supported congregate housing as their preferred living environment. We hypothesized that SOPT would reduce depressive symptoms in senior living communities, and that the effect would be comparable in both the assisted and independent living groups. However, that was not what we found. Our LMEM models of the PHQ-9 scores and categorical levels, as well as the dichotomous indicators of PHQ-9 scores and examination of unadjusted means and percentages, all indicated significantly worse outcomes among those in the assisted living group exposed to SOPT. The means by which SOPT adversely influenced depression among those in assisted living is unclear, and our research study was not designed to explain the underlying mechanism by which SOPT affected depression outcomes, in either positive or negative ways.

A seemingly logical explanation based on “reactive depression” might be that assisted living participants became depressed as they realized their processing speed deficits. However, this appears unlikely for two reasons. First, the SOPT software adjusts its speed based on user performance, meaning that it slows down until the individual user is consistently successful in identifying the targets (car or truck, then location of the Route 66 sign). That is, the design of the SOPT allows users to “start where they are” and improve at their own rate, which in turn should minimize risks of frustration or a sense of failure. Second, the SOPT intervention improved UFOV scores equally for those in both the assisted and independent living SOPT group, as reported previously [32].

Our findings are perhaps best understood in the context of emerging research about causal pathways between depression and cognitive dysfunction [19]. That is, considerable evidence supports the depression-cognitive impairment relationship, including the association of late life depression symptoms with general cognitive function, memory, attention and executive function, and processing speed. The mechanisms of action of the depression-cognitive impairment relationship are not yet understood, although several studies provide important perspectives about possible interactions.

First, a recent study found that low mood was associated with strong activity in a brain subnetwork driven by ß-frequency crosstalk between the brain’s amygdala, which mediates fear and other emotions, and the hippocampus, which aids memory [20]. When present, this amygdala-hippocamus ß-frequency network consistently predicted worsening mood. Second, a recent review of 39 studies that sought to determine if baseline cognitive functioning could predict treatment outcomes of depression treatment [19] concluded that executive dysfunction, particularly in elderly samples, predicted poor response to treatment. Third, the role of executive function in late life depression, and more specifically, processing speed that is a component of executive function, has been implicated in numerous clinical trials [17–19]. Using data from a study on vascular depression, Sheline et al. found that (a) depression severity, age, education, race and vascular burden were each associated with cognitive domains of working memory, executive function, and processing speed in late life depression; and (b) slower processing speed appeared to be the core cognitive deficit in late life depression, followed by executive function [21]. Processing speed was also central to the effect that depression and executive function had on functional impairment among older people with mild cognitive impairment (MCI) [14]. And in a large longitudinal study, presence of comorbid depression symptoms increased the risk of both onset of amnestic MCI, and amnestic MCI progressing to dementia [15]. Thus, there are many potential opportunities for the demands of SOPT to impact on depression in a positive way (as observed in earlier clinical trials), but also for age-related cognitive changes and existing mild depression to interact in a negative way.

Participants in the assisted living group exposed to SOPT had significantly slower (183.8 *ms*) UFOV scores at baseline and significantly higher baseline levels of depressive symptoms (PHQ-9 scores ≥ 10 for 12.7% vs. 3.8%). Taken together, the processing speed of the assisted living participants exposed to SOPT may have been too low, and their depression levels too high, for depression to be beneficially affected by this intervention. The neurobiological pathway between depression and cognitive speed of processing may have been mediated by a variety of characteristics, traits, or health conditions that elevate risk for needing assisted living.

Of note, chronological age was not significantly different between participants in assisted and independent living groups. Thus, the “null” effect of the SOPT intervention on depression levels (i.e., no significant positive or negative effect) among independent living participants, and their generally higher UFOV performance is likely not a function of chronological age itself. Instead, currently unidentified differences in the characteristics of these groups may explain factors that lead to worsening depression among assisted living participants assigned to the SOPT intervention.

Additional research is needed to develop explanatory models of underlying pathways between cognitive speed of processing and depression, and to conduct clinical trials that clarify the mechanism of action and factors that cause and contribute to depression outcomes. Until additional research illuminates the findings reported here, we suggest that SOPT, and particularly *Road Tour/Double Decision*, should not be used among seniors who reside in assisted living settings, and that those in independent living who may use the software should be carefully monitored related to depression levels. Furthermore, encouragement of “brain health” initiatives and games in senior living settings should be carefully evaluated for possible untoward effects, including but not limited to increasing levels of depression.

### Limitations

Like many RCTs, our study was not without limitations that may have influenced depression outcomes. We included senior living communities in only one rural midwestern state and our sample size was modest. Methods to gather time on task in the attention control group proved ineffective due to poor fidelity on the part of the Study Liaisons. Completed time on task in the SOPT groups was substantially lower than the recommended dose, and statistically lower in the assisted living SOPT group. We did not include a measure of cognitive function that would have provided additional insights about potential mild impairment in the sample that may have influenced their level of participation and performance. We also did not evaluate participants’ views about the acceptability of, or their satisfaction with the computerized ‘“games” that may have interacted with their mood. Stienermen’s comments related to technology-enabled cognitive training are also relevant here, such that group level means obscure the relative contribution of nonresponders and those with robust changes which suggests that mediating factors like personality and motivation may help inform individualized predictors of training response [49].

## Supporting information

S1 Protocol Improving mood research protocol. (DOCX)

S1 Dataset 274 participants with complete baseline and 12 month data. (XLS)

S1 Checklist CONSORT 2010 checklist—Randomized trial. (DOC)
