# Supplementary material for: Speed of processing training and depression in assisted and independent living: A randomized controlled trial
Source: PLoS One. 2019 Oct 17;14(10):e0223841. doi: 10.1371/journal.pone.0223841 (PMC6797094; doi:10.1371/journal.pone.0223841)
Supplement: S1 Protocol — (DOCX) [file pone.0223841.s001.docx]

**Improving Mood Research Protocol**

**Title:** *Improving Mood in Assisted Living Settings Using a Cognitive Training Intervention*

**PI:** Marianne Smith, PhD, RN

**Address:** University of Iowa, College of Nursing; 50 Newton Road, CNB; Iowa City, IA 52242

**Email:** Marianne-smith@uiowa.edu

**Ethics Approval and Informed Consent**

Institutional Review Board approval of the study protocol was obtained through the university committee for biomedical research (IRB-01), #201208786. This committee has provided continuous oversight and monitoring. The research protocol was registered at ClinicalTrials.gov (NCT 01763216) on January 3, 2013 (before any participants were approached for enrollment) and was updated throughout the study. Written informed consent was obtained from all participants.

**Funding**

This study was funded by the National Institute for Nursing Research Award # NIH R01-NINR13908. Posit Science Corporation provided free copies of *Road Tour* and *Double Decision* for use by participants but had no role in the design and conduct of the study, or any manuscripts submitted for publication. Licenses for *Boatload of Crosswords* were purchased from Boatload Puzzles, LLC using NINR grant funds.

**Materials and Methods**

The *Improving Mood* study was a 5-year, two arm, parallel randomized controlled trial (RCT) that compared the speed of processing training program known as *Road Tour* to attention control using computerized crossword puzzles in assisted living (AL) and related senior living settings. Three specific aims evaluated the effect of *Road Tour* vs. attention control on: (1) cognitive processing speed; (2) reducing depressive symptoms and the risk of onset of suspected clinical depression; and, (3) depression-related comorbidities including anxiety, pain, and health-related quality of life among older adults living in AL.

***Setting recruitment and engagement***

A community-based research approach was used to engage AL settings as partners in conducting the research project. The goal was to engage up to 30 AL settings that would each recruit a minimum of 10 older adult participants to achieve the target of 300 total participants. As partners in the research, each organization identified one or more staff members who completed university-sponsored, online human subjects training and acted as onsite research assistants (RAs). These onsite RAs, called Study Liaisons, were trained to recruit, consent, and train participants to use the computer games, conduct computer-based assessments, and facilitate telephone assessments conducted by university team members. Study Liaison preparation used detailed training manuals and fidelity checks performed by the Study Coordinator to assure the integrity of study procedures at partner sites.

Adaptation: Although AL programs were plentiful, the vast majority were too small to identify 10 participants who would participate, and/or didn’t have staff resources to support the participatory study approach. Moreover, senior living leaders questioned the logic of involving only AL residents, noting that many residents on the same campus were “one service away” from being classified as “assisted” based on their age, health, and function. Others indicated that they had abandoned policies that required residents to relocate when they needed services. In turn, many older adults living in housing that was defined as “independent living” (IL) on their campus were receiving personal assistance similar to those living in housing defined as AL. As a result, older adults residing in IL apartments on senior living campuses whose AL services were also involved in the study were also invited to participate.

***Study participants***

All older adults living at partner sites were invited to enroll to maximize generalizability. Inclusion/exclusion criteria included being 60 years old or older, residing in a participating AL, speaking English (the language of *Road Tour*), and self-report having sufficient visual acuity (with glasses) to use a computer, physical ability to operate a computer keyboard and mouse, and capacity to provide informed consent. Written informed consent was obtained by Study Liaisons at each site using standard procedures that were modeled by university team members to assure both comfort with the process and fidelity to the methods.

Adaptation. We adjusted the lower boundary of our inclusion criteria to 55 years to conform to admission policies in partner settings. This adjustment allowed the Study Liaisons to avoid a sense of discrimination against slightly younger adults living in their communities, and greatly simplified their advertising and recruitment approaches.

***Experimental Design***


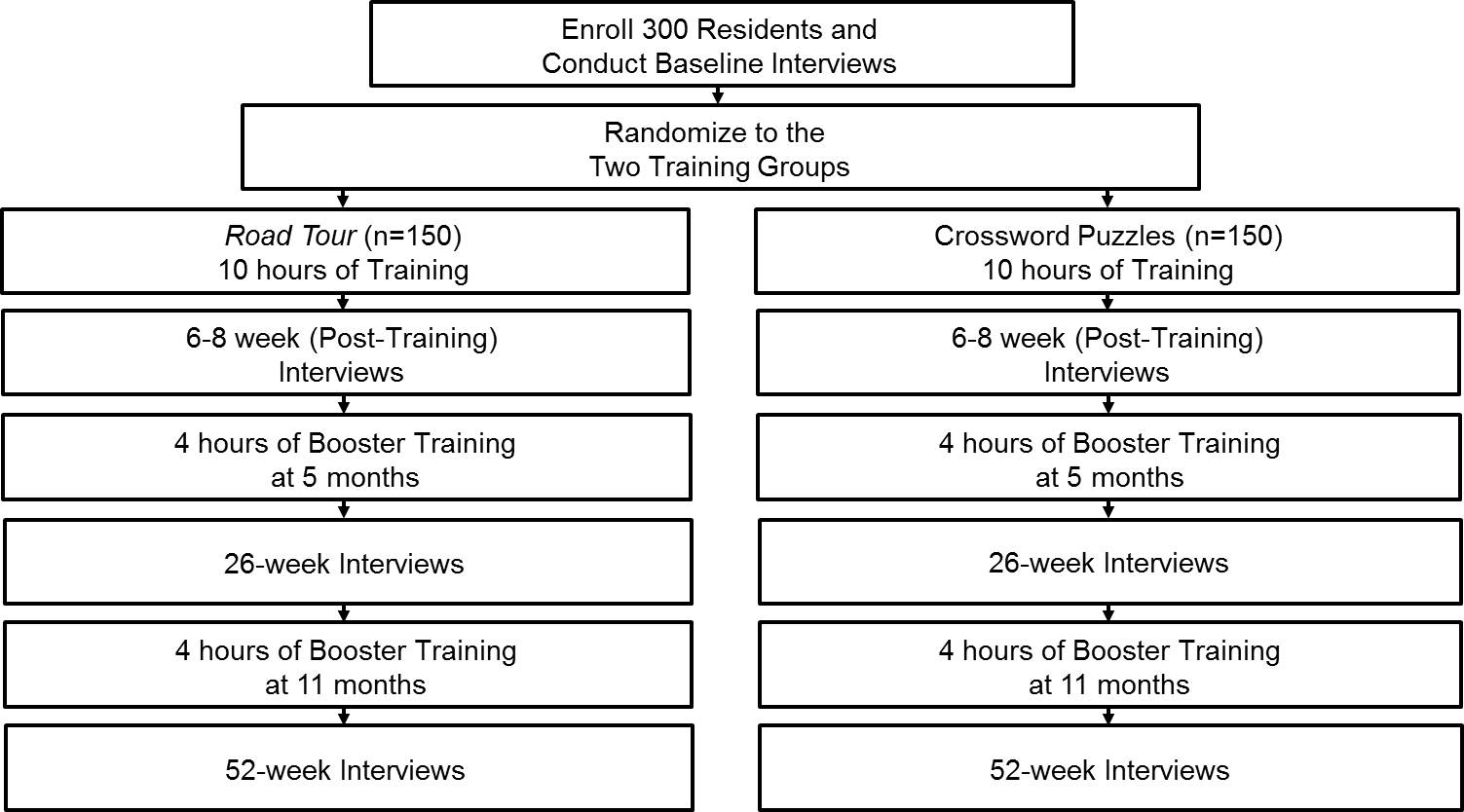


Eligible older adults who completed informed consent with Study Liaisons were scheduled for baseline telephone interviews conducted by university team members. All primary outcome measures were collected via telephone interviews, except for the speed of processing measure (Useful Field of View, UFOV) which was collected onsite by Study Liaisons. Randomization occurred after baseline interviews were completed. Participants were asked to complete 10 hours of training during the first 5-6 weeks, and 4 additional hours of training at 5 and 11 months for a total targeted dose of 18 hours.

**Randomization.** Participants were randomized to training groups using block randomization within sites and types of care (AL and IL) to maintain balance across treatment conditions by senior living community and residence type (AL and IL). The study statistician determined the order of assignments using a computer-generated list of random numbers using block sizes of two and four. Participant IDs and assignments were concealed in sequentially numbered opaque envelopes stored in separate bins (one for each site) kept in a locked cabinet within the Study Coordinator’s office.

***Assessment and Data Management***

Computer-assisted telephone interviews (CATI) were used to assess participant characteristics and outcome measures at baseline, and outcome measures again at post-training (6-8 weeks post-randomization), and at 26 and 52 weeks post-randomization. The speed of processing assessment was administered at the four time points by Study Liaisons using study computers at partner sites. Usage information embedded in the intervention software was sent to research investigators over the internet using secure file encryption protocols. This included the number of minutes played, number of sessions, and the percentage of completing all available exercise sets.

All data collection was monitored by the Study Coordinator. Standard procedures were used for programming CATI software, interviewer training, data entry and editing, as well as database management, updating, reconciliation, data retrieval and statistical computing, and data security and confidentiality. Codes were written so that missing and implausible values were automatically flagged in real time during the interviews for verification when entered. Onsite speed of processing assessments were saved using participant ID numbers and copied to encrypted flash drives that were mailed to the Study Coordinator. Results were printed first and then entered electronically, with the printed originals kept in secure storage. All data were stored on a secure university server that is backed up daily and uses a three-tiered authorization and strong password system to protect data.

***Instruments and Measures***

**Speed of Processing Measures (Aim 1).** Speed of processing was measured using the *Useful Field of View (UFOV)* PC mouse version. The *UFOV* is well-established, has high reliability and validity, and is the gold standard for measuring visual processing speed.^1^ It is computer administered and takes 5 to 7 minutes to complete. Three subtests, stimulus identification, divided attention, and selective attention, are each automatically scored from 17-500 miliseconds (ms) reflecting the shortest exposure time at which the participant could correctly perform each subtest 75% of the time. A composite outcome score of the three subtests ranges from 51-1500 ms, with lower scores reflecting faster speed of processing. Normative data for 2759 participants aged 65 to 94 years in the *ACTIVE* clinical trial indicates that the baseline UFOV composite scores ranged from 86 to 1500 ms, with a mean of 481.9 ms and standard deviation of 247.5 ms.^2^

**Depression Symptoms (Aim 2).** The frequency and severity of depression symptoms were assessed using two self-report scales that were used at each time point. The 12-item version of the Centers for Epidemiological Studies Depression (CESD) scale^3^ is a shorter version of the original CESD-20^4^ that has been a gold standard for screening for depression in epidemiological and public surveys, and has very high reliability and validity. The CESD-12 was used in both *ACTIVE* and *IHAMS* trials which allows comparison of depression outcomes among participants in this study and subjects in earlier studies. CESD-12 is composed of short statements scored from 0=rarely or none of time (less than 1 day per week) to 3=all of the time (5-7 days per week) for a score range of 0-36, and has a cut-off score of >9 for suspected clinical depression.^5-7^

The 9-item Patient Health Questionnaire (PHQ-9)^8^ also has very high reliability and validity, is widely used in both research and clinical settings, and performs well in telephone assessments.^9,10^ The PHQ-9 items reflect the nine DSM-5 diagnostic criteria for major depression^11^ and are rated from 0=not at all to 3=nearly every day for a total score range of 0-27. Established cut-points are: 0-4=minimal depression; 5-9=mild depression; 10-14=moderate depression; 15 or greater = severe depression. Scores >10 indicate clinically significant depression.^12^ The PHQ-9 directly assesses two hallmarks of major depression (prominent dysphoria and anhedonia) and is described in a large body of late life depression research.

**Depression-Related Health Outcomes (Aim 3).** Health-related quality of life (HRQoL), pain and anxiety measures used in the study have well-established reliability and validity and age-specific national norms for comparative purposes. HRQoL was measured using the SF-36 Health Survey^13^ at baseline and 52 months to allow direct comparison of one-year effects with *IHAMS* and *ACTIVE* studies. The shorter SF-12^14^ was used at 6-8 and 26 weeks to approximate SF-36 scores at these intermediate assessments while minimizing respondent burden. Both the SF-12 and SF-36 include the standard single item self-rated health question. Pain was assessed using the Brief Pain Inventory (BPI) that was developed for assessing cancer pain, validated for use in general populations, and is widely used.^15-17^ The BPI includes four pain severity and seven pain interference items that are rated from 0 to 10, an open-ended pain severity item that is rated 0 to 10, two open-ended questions about use of medications and treatments used to treat pain, and one question about pain relief from treatments that is rated 0 to 10. Anxiety symptoms were assessed using the 7-item Generalized Anxiety Disorder (GAD-7) scale^18^ that rates the DSM-IV^19^ criteria for GAD on a scale from 0=not at all to 3=nearly every day. Cut-points for the GAD-7 are comparable to the PHQ-9, with 0-4= minimal anxiety; 5-9=mild anxiety; and >10=clinically significant anxiety.

**Covariates.** Personal and health-related characteristics were collected at baseline. Personal characteristics included: sociodemographics (age, race, sex, marital status, education, and income), social support (5 items scored on a 5-point scale from 0=none to 4=all of the time), and presence of common chronic conditions like high blood pressure, arthritis, heart disease, diabetes, lung disease, as well as anxiety disorder, depression and current use of depression treatments. Characteristics of partner organizations included number of older adults served, type of programs and services provided, type and number of staff employed, profit/ownership status, and years in operation.

***Intervention and Attention Control Training Programs***

**Intervention.** *Road Tour* is the second-generation speed of processing training program that was evaluated in *ACTIVE* (in its MS-DOS format) and used in *IHAMS* (in its MS Windows format). The training was designed to improve the efficiency and accuracy of visual information processing and the ability to perform complex visual attention tasks. It focuses on improving the speed and accuracy with which users identify and locate visual information using a divided attention format. Over time, the difficulty and complexity of each task is systematically increased as users attain specified performance criteria. Difficulty is increased by reducing visual stimuli duration, adding visual distracters, increasing similarity between target and distracter stimuli, and presenting visual targets over a broader spatial expanse.

To initiate training, the user clicks on the start button. One of two vehicles, either a car or a truck, appears in the center of screen. Seven distracter stimuli (rabbit crossing signs) and the target sign (Route 66) appear in the periphery. The stimuli (car vs. truck, and rabbit crossing vs. Route 66 sign) are presented for a specified time and then disappear briefly. A car and truck appear, and the user clicks on the correct target vehicle (car or truck). Then the user selects the circular location where the Route 66 sign had appeared. The training program is tailored to the participant’s performance by maintaining a 75% success rate before increasing the challenge level. As the user progresses, three changes increase the task difficulty: the visual field expands; an increasing number of distracters appear; and the vehicle pairs morph to become more similar and thus more difficult to differentiate.

Adaptation. Posit Science Corporation’s CD-ROM based *Road Tour* training program was used until support was unexpectedly discontinued in August 2014, roughly 17 months after starting enrollment. The web-based replacement, *Double Decision*, (<https://www.brainhq.com/why-brainhq/about-the-brainhq-exercises/attention/double-decison>) was used from 2014 through the end of enrollment in 2015. The training features of *Double Decision* are the same as *Road Tour* (as described above). The transition to *Double Decision* presented short-term delays as we developed new recruitment and training materials for *Double Decision* and negotiated support for participants already using *Road Tour* to assure use of one format or the other, but not both by a single participant.

**Attention Control.** *Boatload of Crosswords* (<http://www./boatloadpuzzles.com>) served as the attention control condition. *Boatload of Crosswords* is one of the oldest and most popular crossword puzzle games commercially available. The software offers the user a choice between three puzzle sizes, three levels of complexity, and varying font sizes. *Boatload of Crosswords* also provides optional help features, like filling in letters and words, showing incorrect letters or words in red, and solving the puzzle, that minimize frustration levels often associated with puzzle completion. *Boatload of Crosswords* was chosen for this study because, like *Road Tour*, it is computerized, very popular and easy to use; many older adults enjoy doing crossword puzzles; and it was successfully used as the attention control condition in *IHAMS*.^6^

***Approach***

The community-based approach first engaged senior living programs and services who agreed to (a) having a dedicated a staff member act as the on-site RA (Study Liaison), and (b) recruit a minimum of 10 older adults in their setting to participate in the study. Required documents for subawards were completed, signed and filed with the University Department of Sponsored Programs. Staff member(s) identified as Study Liaisons were trained and certified onsite by the Study Coordinator or her designee using university-endorsed Certifications in Human Subjects Protections (CITI) training program (<https://hso.research.uiowa.edu/certifications-human-subjects-protections-citi>). Each partner was provided two (or more) computers that were programmed specifically for study use, including fields for participants to log-on and use their assigned software, and also administration of the UFOV by Study Liaisons. The Study Coordinator and/or designated university research assistants trained Study Liaisons to (a) use *Road Tour/Double Decision* and *Boatload of Crosswords* software using detailed program materials developed for use in the study; and (b) conduct informed consent with participant, including pre-screening for inclusion criteria and capacity to sign meaningful informed consent. An individualized manual was developed for each partner that outlined (a) key contacts in the partner organization and the university research team; (b) standard procedures for study implementation, including informed consent, telephone interview methods and timing, secure storage of informed consents and UVOF data until picked up by the university research team, and management of questions by participants; and (c) suicide management protocols (given that the PHQ-9 contained a self-harm question). Study Liaison were provided practice time related to software use, and supported by university team members when conducting informed consent with their first three (minimum) participants, when providing *Road Tour* and *Puzzles* training to their first 3-4 participants (assuring support and assistance for each of the two program and sufficient repetition to be comfortable), and when administering the on-site computerized UFOV test.

Computer-assisted-telephone-interviews (CATI) at baseline post-training, 26 weeks ad 52 weeks were scheduled and monitored by the Study Coordinator, and were conducted by specially training university research team members (RAs). Telephone interviews were scheduled to fit the personal preferences and routines of individual participants. Each interview began by rechecking for comfort related to the time of day and any unexpected demands. Potential for fatigue related to the length of the interviews (30 to 35 minutes each) was be monitored by the interviewer. Warning signs of fatigue, such as audible yawning or inattentiveness, served as cues to ask the participant if he/she would like to take a short break, or suspend the interview and resume it later on. Supportive materials to advance successful completion of interviews included use of appointment cards for participants, reminders by Study Liaisons, Answer Guides that helped participants follow answer choices by scale, and partners-specific procedures for missed calls to assure smooth and timely data collection. CATI data was entered using the university-supported Research Electronic Data Capture (REDCap) management system (<https://its.uiowa.edu/redcap>). The data entry process was coded so that all items offered “pass” options and all items in one sections must be completed before moving to the next.

The CATI system operated from the highly secure College of Public Health main, multi-RAID SQL relational database server. This SQL server uses a three-tiered security system to restrict access and protect data. These steps require (1) ITS-issued and approved ID logins, (2) a strong, quarterly-changing 15-character password in conjunction with prior access authorization for the specific SQL server directories in question, and (c) specified prior approvals for read, write, and other privileges. The SQL server is automatically backed-up every night to a second multi-RAID SQL server. All SQL servers are staffed, maintained, and monitored by ITS staff 24/7.

***Analysis***

Descriptive statistics including Student’s t-tests and chi-squared analysis are used to compare the intervention and attention control groups at baseline, and to examine differences based on living setting (AL vs. IL). The original analysis plan was to evaluate the three specific aims using each person serving as his/her own control in linear panel analyses ^20^ with GEE to correct for clustering within sites, and, logistic regression GEE models for binary outcomes. Power estimates were based on the results from the earlier *IHAMS* and *ACTIVE* studies. Setting a one-tailed *α* at 0.05 and allowing for 10% attrition, we estimated > 80% power to detect the improvements in *UFOV*, 73% power to detect changes in depressive symptoms and 68% power to detect suspected onset of clinical depression.

Adaptations. Because of the inclusion of participants from IL settings, the analytic approach will be altered. We will use random effects general linear mixed models. The dependent variable will be the outcome of interest at the time point of interest. The clustering (random effects) variable will be the AL or IL from each participating senior living community. The two main factors will be intervention vs. attention control and AL vs. IL. The interaction between these two factors will be included to test for heterogeneity of treatment effects (HTEs) between AL vs. IL. Covariates will include the outcome of interest at baseline, age, and sex.

Two *ad hoc* analyses for the first specific aim will also be conducted. The first will involve expanding the intervention vs. attention control from a binary indicator to a trichotomous indicator of *Road Tour* vs. *Double Decision* vs. attention control to test for potential HTEs between the two speed of processing platforms. The second will involve opportunistic dosing testing based on whether the intervention participants completed eight or more hours of intervention training vs. less than eight hours.

References

1. Edwards JD, Vance DE, Wadley VG, Cissell GM, Roenker DL, Ball KK. Reliability and validity of useful field of view test scores as administered by personal computer. *J Clin Exp Neuropsychol.* 2005;27(5):529-543.

2. Edwards JD, Ross LA, Wadley VG, et al. The useful field of view test: normative data for older adults. *Arch Clin Neuropsychol.* 2006;21(4):275-286.

3. Kohout FJ, Berkman LF, Evans DA, Cornoni-Huntley J. Two shorter forms of the CES-D (Center for Epidemiological Studies Depression) depression symptoms index. *Journal of aging and health.* 1993;5(2):179-193.

4. Radoff LS. The CES-D Scale: A self-report depression scale for research in the general population. *Appl Psychol Meas.* 1977;1(3):385-401.

5. Wolinsky FD, Mahncke HW, Weg MW, et al. The ACTIVE cognitive training interventions and the onset of and recovery from suspected clinical depression. *J Gerontol B Psychol Sci Soc Sci.* 2009;64(5):577-585.

6. Wolinsky FD, Vander Weg MW, Howren MB, Jones MP, Dotson MM. The effect of cognitive speed of processing training on the development of additional IADL difficulties and the reduction of depressive symptoms: results from the IHAMS randomized controlled trial. *Journal of aging and health.* 2015;27(2):334-354.

7. Wolinsky FD, Vander Weg MW, Martin R, et al. The effect of speed-of-processing training on depressive symptoms in ACTIVE. *J Gerontol A Biol Sci Med Sci.* 2009;64(4):468-472.

8. Kroenke K, Spitzer RL, Williams JB. The PHQ-9: validity of a brief depression severity measure. *J Gen Intern Med.* 2001;16(9):606-613.

9. Fine TH, Contractor AA, Tamburrino M, et al. Validation of the telephone-administered PHQ-9 against the in-person administered SCID-I major depression module. *J Affect Disord.* 2013;150(3):1001-1007.

10. Pinto-Meza A, Serrano-Blanco A, Penarrubia MT, Blanco E, Haro JM. Assessing depression in primary care with the PHQ-9: can it be carried out over the telephone? *J Gen Intern Med.* 2005;20(8):738-742.

11. American Psychiatric Association. *Diagnostic and Statistical Manual of Mental Disorders, Fifth Edition (DSM-5).* Arlington, VA: American Psychiatric Association; 2013.

12. Manea L, Gilbody S, McMillan D. Optimal cut-off score for diagnosing depression with the Patient Health Questionnaire (PHQ-9): a meta-analysis. *CMAJ.* 2012;184(3):E191-196.

13. Beusterien KM, Steinwald B, Ware JE, Jr. Usefulness of the SF-36 Health Survey in measuring health outcomes in the depressed elderly. *J Geriatr Psychiatry Neurol.* 1996;9(1):13-21.

14. Jenkinson C, Layte R, Jenkinson D, et al. A shorter form health survey: can the SF-12 replicate results from the SF-36 in longitudinal studies? *J Public Health Med.* 1997;19(2):179-186.

15. Atkinson TM, Rosenfeld BD, Sit L, et al. Using confirmatory factor analysis to evaluate construct validity of the Brief Pain Inventory (BPI). *J Pain Symptom Manage.* 2011;41(3):558-565.

16. Cleeland CS, Ryan KM. Pain assessment: global use of the Brief Pain Inventory. *Ann Acad Med Singapore.* 1994;23(2):129-138.

17. Cleeland CS. *The Brief Pain Inventory User User Guide.* Houston, TX: The University of Texas M. D. Anderson Cancer Center;2008.

18. Spitzer RL, Kroenke K, Williams JB, Lowe B. A brief measure for assessing generalized anxiety disorder: the GAD-7. *Arch Intern Med.* 2006;166(10):1092-1097.

19. American Psychiatric Association. *Diagnostic and Statistical Manual of Mental Disorders, Fourth Edition (DSM-IV).* Arlington, VA: American Psychiatric Association; 2000.

20. Kessler RC, Gerrenberg DF. *Linear Panel Analysis.* New York, NY: Wiley; 1981.
